# First Report of Okadaic Acid and Pectenotoxins in Individual Cells of *Dinophysis* and in Scallops *Argopecten purpuratus* from Perú

**DOI:** 10.3390/toxins10120490

**Published:** 2018-11-23

**Authors:** Alex Alcántara-Rubira, Víctor Bárcena-Martínez, Maribel Reyes-Paulino, Katherine Medina-Acaro, Lilibeth Valiente-Terrones, Angélica Rodríguez-Velásquez, Rolando Estrada-Jiménez, Omar Flores-Salmón

**Affiliations:** Organismo Nacional de Sanidad Pesquera (SANIPES), Av. Domingo Orué N° 165, Surquillo Lima 34, Peru

**Keywords:** okadaic acid, pectenotoxins, *Dinophysis*, *D. acuminata*-complex, *D. caudata*, *Argopecten purpuratus*

## Abstract

Causative species of Harmful Algal Bloom (HAB) and toxins in commercially exploited molluscan shellfish species are monitored weekly from four classified shellfish production areas in Perú (three in the north and one in the south). Okadaic acid (OA) and pectenotoxins (PTXs) were detected in hand-picked cells of *Dinophysis* (*D. acuminata*-complex and *D. caudata*) and in scallops (*Argopecten purpuratus*), the most important commercial bivalve species in Perú. LC-MS analyses revealed two different toxin profiles associated with species of the *D. acuminata*-complex: (a) one with OA (0.3–8.0 pg cell^−1^) and PTX2 (1.5–11.1 pg cell^−1^) and (b) another with only PTX2 which included populations with different toxin cell quota (9.3–9.6 pg cell^−1^ and 5.8–9.2 pg cell^−1^). Toxin results suggest the likely presence of two morphotypes of the *D. acuminata*-complex in the north, and only one of them in the south. Likewise, shellfish toxin analyses revealed the presence of PTX2 in all samples (10.3–34.8 µg kg^−1^), but OA (7.7–15.2 µg kg^−1^) only in the northern samples. Toxin levels were below the regulatory limits established for diarrhetic shellfish poisoning (DSP) and PTXs (160 µg OA kg^−1^) in Perú, in all samples analyzed. This is the first report confirming the presence of OA and PTX in *Dinophysis* cells and in shellfish from Peruvian coastal waters.

## 1. Introduction

Diarrhetic shellfish poisoning (DSP) toxins cause a gastrointestinal human health syndrome with the main symptoms being nausea, diarrhea, vomiting, and gastrointestinal pain [1,2]. Okadaic acid (OA) and its congeners, dinophysistoxins (DTX1, DTX2), their high-polarity precursors (DTX4, DTX5), and their 7-*O*-acyl-derivatives (“DTX3”) are liposoluble polyethers that have been designated as diarrhetic shellfish toxins [3,4,5]. Pectenotoxins are liposoluble, non-diarrheogenic, polyether lactones which may co-occur with DSP toxins and can be coeluted with them [6] using the usual extraction procedures. The two groups of toxins have been found in different species of *Dinophysis* (*D. acuminata*, *D. acuta*, *D. caudata*, *D. fortii*, *D. infundibula*, *D. miles*, *D. norvegica*, *D. ovum*, *D. sacculus*, *D. tripos*) and two species of *Phalacroma* (*P. mitra*, *P. rotundatum*) [5]. In addition, OA and its congeners have been found in several benthic species from the genus *Prorocentrum* (*P. concavum*, *P. texanum*, *P. arenarium*, *P. lima*) [5,7,8].

Filter-feeding bivalves accumulate algal toxins and are the main vectors transferring them to humans through the food web. DSP toxins and pectenotoxins (PTXs) pose a global threat to public health and aquaculture [9,10,11,12]. The analysis of DSP toxins for monitoring programmes in Perú have been carried out only by mouse bioassay [13], and there is no information on the toxin profiles of either the potentially toxic species of *Dinophysis* or of the contaminated molluscan shellfish. Nevertheless, *D. acuminata*, *D. caudata*, *D. tripos*, and *Phalacroma rotundatum* (=*Dinophysis rotundata*) have been reported in Peruvian coastal waters [14], and their toxins associated with positive results for lipophilic toxins in mouse bioassays [13]. There have also been reports on the occurrence of the benthic dinoflagellate *Prorocentrum lima* [13], but to date this species has not been associated with DSP events in Perú.

**Figure 1:**
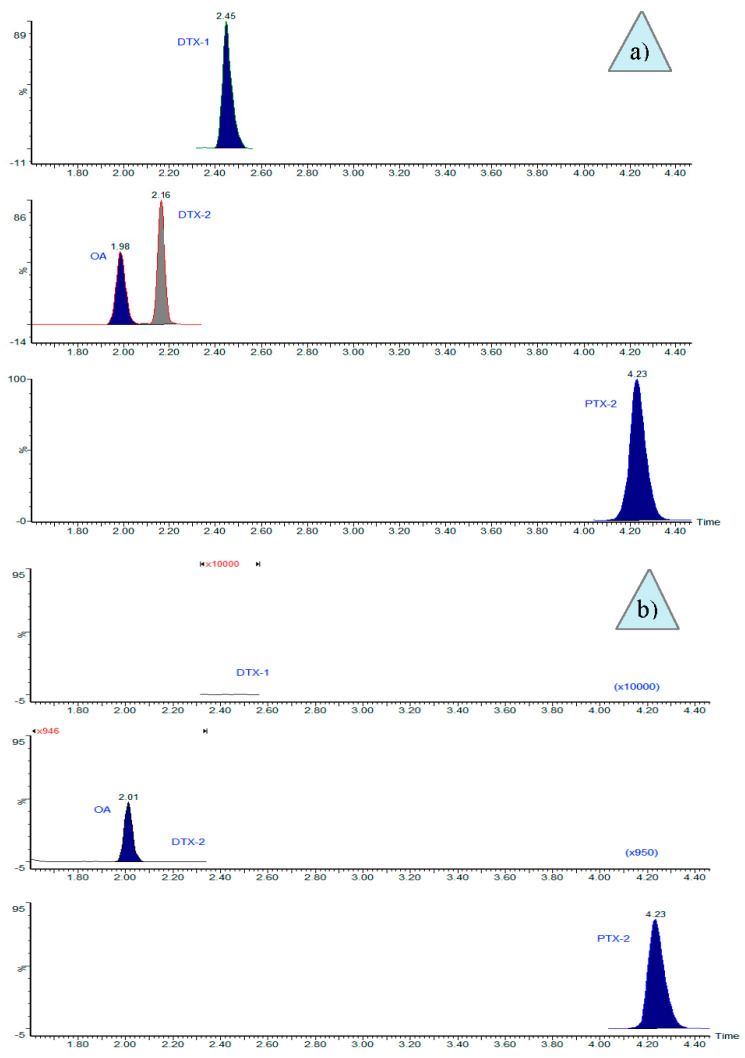
Selective ion chromatograms from the LC-MS/MS analyses of isolated cells of *Dinophysis acuminata*-complex in: (**a**) multitoxin standard, 3 ng mL^−1^; and (**b**) chromatogram of the sample from Bahía de Sechura-Vichayo, where the absence of DTX1 (increase ×10,000) and DTX2 (increase ×950) was observed.

During 2017 and 2018, seawater and shellfish samples were collected from classified shellfish production areas (Figure 1), in the framework of the Molluscan Shellfish Safety Programme (PCMB) of the National Fisheries Health Organization of Perú (SANIPES), to establish the relationship between the toxic profiles detected in shellfish and the occurrence of potentially toxic dinoflagellates.

## 2. Results

Chromatograms from the LC-MS/MS analyses of hand-picked cells showed that the toxin profile of *Dinophysis acuminata*-complex cells from the northernmost production area (Sechura Bay) included OA and PTX2 (Figure 1b).

Other toxins, such as DTX1 and DTX2, were not found (LOD = 0.16 ng mL^−1^). In contrast, only PTX2 was detected in the analyses of picked cells of *Dinophysis* cf. *acuminata* and *D. caudate* from the other three areas (i.e., Independencia Bay, Samanco Bay, and Salinas), with an average toxin content of 9.3 pg PTX2 cell^−1^ (Table 1).

The same profile of toxins present in the cells was detected in the shellfish *Argopecten purpuratus* from the same production areas (Table 2).

## 3. Discussion

Okadaic acid and PTX2 were detected in both *Dinophysis* cells and scallops. In some cases, both toxins were present but in others only PTX2 was detected. There are previous reports on *Dinophysis* species with a toxin profile constituted by only PTX2. That was the case with *D. acuminata* cells isolated from Inglesa Bay [15] and from Reloncaví estuary [4], both from Chile, as well as with *D. caudata* from the Galician Rías Bajas, northwest Spain [16] and *D. acuminata* from Danish waters [17]. Likewise, our analyses of shellfish meat from the same areas in Perú were in agreement with the toxin profiles of the dinoflagellates, that is, just PTX-2 (10.3–22.2 µg PTX2 kg^−1^ meat) and no traces of OA in the areas where *Dinophysis* species had the same profile.

The Peruvian strains of *Dinophysis* seemed to contain much lower amounts of toxin per cell than those reported from Galicia, Spain (*D. caudata*: 100.0–127.4 pg PTX2 cell^−1^) [16] and from Chile (*D. acuminata*: 180 pg PTX2 cell^−1^), which had a toxin content one order of magnitude higher than the Peruvian strains (Table 2). 

Cells of *Dinophysis acuminata*-complex around Sechura Bay showed a higher variability in their PTX2 content, with values ranging from 1.5 to 11.1 pg cell^−1^ and to a lesser extent in their OA cell quota (from 0.3 to 0.8 pg cell^−1^). We do not know if this variability is due to the co-occurrence of different species of the *D*. cf. *acuminata* in the same area. Nevertheless, previous laboratory experiments and field data have shown a large variability of toxin content per cell of *Dinophysis* associated with different phases of the population growth and their interaction with environmental conditions [18]. More studies, including physiological and genetic factors affecting toxin profiles and content, are needed to clarify these questions. The presence of OA and PTX2 has been also reported in *D. acuminata* from Lake Orbetello, Italy [19] and in New Zealand [20], USA [4], and Japan [21], where other toxins (e.g., DTX1 and some PTX analogues) were also reported.

*D. caudata* from the same bay had 5.8 pg PTX2 cell^−1^. Therefore, there was a co-occurrence of two toxic *Dinophysis* species in this area. Scallop samples (“concha de abanico”) during the occurrence of these species reached toxin levels ranging from 7.7 to 15.2 µg OA kg^−1^ and from 10.7 to 34.8 µg PTX2 kg^−1^ (Table 2).

The low toxin content found in *Dinophysis* cells in Perú suggests a low risk of DSP toxins accumulation in shellfish above the regulatory levels. That was the case during the 2 years (maximum levels in Independencia Bay: <LOD OA and 22.2 µg kg^−1^ PTX2; Samanco Bay: <LODOA and 16.4 µg kg^−1^ PTX2; Salinas: <LOD OA and 12.2 µg kg^−1^ PTX2; Sechura Bay-Puerto Rico: 10.4 µg kg^−1^ OA and 48.2 µg kg^−1^ PTX2; Sechura Bay-Barrancos: 8.6 µg kg^−1^ OA and 21.0 µg kg^−1^; Sechura Bay-San Pedro: 15.2 µg kg^−1^ OA and 43.7 µg kg^−1^ PTX2; and Sechura Bay-Las Delicias: 7.7 µg kg^−1^ OA y 19.6 µg kg^−1^, June 2016–May 2018) of toxin monitoring of scallops, *A. purpuratus*, by LC-MS/MS. During this period, toxin levels never reached regulatory limits, although *Dinophysis* densities above 10^4^·cells L^−1^ were recorded. *Dinophysis* densities of around 10^3^ cells L^−1^ are considered a bloom, and have often been related to toxic outbreaks in other parts of the world [5]. 

## 4. Conclusions

The toxin profiles, including OA and PTX2, of several species of *Dinophysis* and of scallops (*Argopecten purpuratus*) from shellfish production areas in Perú were characterized. Different species included in the *Dinophysis acuminata*-complex and *D. caudata*, producers of toxins regulated by the EU, co-occur in northern Perú. The toxic species of *Dinophysis* from Sechura Bay, Samanco Bay, Salinas, and Independencia Bay showed low cell-toxin content (pg cell^−1^) in comparison with those reported for the same species in other parts of the world, although more studies, including physiological and genetic factors affecting toxin profiles and content, are needed. *Dinophysis acuminata*-complex and *D. caudate* are most likely the main species concerning molluscan shellfish safety in Perú.

## 5. Materials and Methods

### 5.1. Field Sampling

**Figure 2:**
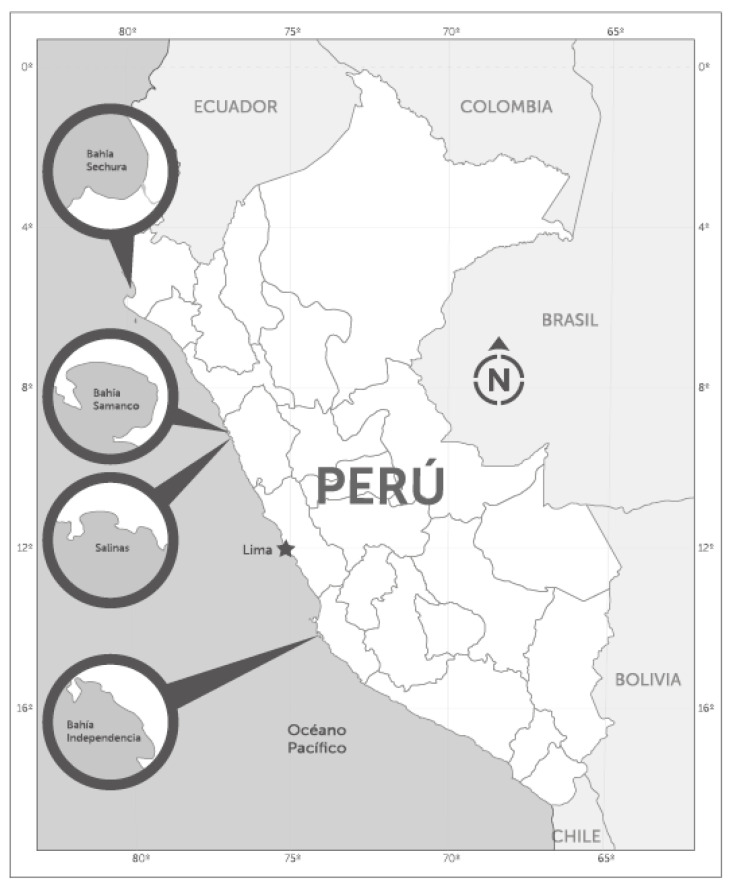
Location of sampling stations.

Seawater and shellfish samples for the analyses of potentially toxic phytoplankton and shellfish toxins were collected weekly in the framework of the National Molluscan Shellfish Safety Programme (PCMB) of the National Fisheries Health Organization of Perú (SANIPES), which is the national competent authority for the control of seafood safety. Samples from classified shellfish production areas were analyzed at the SANIPES official laboratory. During 2017 and the first half of 2018, seawater and scallops (*Argopecten purpuratus* “concha de abanico”) samples were collected at the fixed monitoring stations in Sechura Bay, Samanco Bay, Salinas, and Independencia Bay (Figure 2) for analyses. The objective was to determine the toxin profiles in the plankton and shellfish at the time of detection of lipophilic shellfish toxins. Two kinds of water samples were collected at each station for phytoplankton analyses: (i) vertical net-hauls (10 µm mesh size), with no fixatives added, for the identification of the species in vivo and for single cell isolations; (ii) depth-integrated hose-samples (hose length 15 m), which were immediately fixed with acid Lugol’s solution, for quantitative analyses by the standard Utermöhl method [22].

### 5.2. Single Cell Isolations

**Figure 3:**
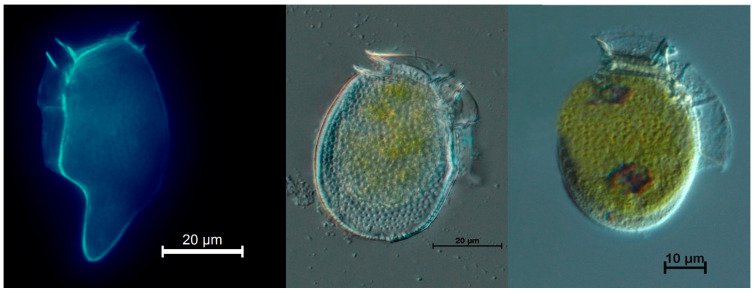
*Dinophysis* cells isolated from Samanco Bay, Perú. (**Left**) Epifluorescence of *Dinophysis caudata*; (**middle**) and (**right**) DIC micrographs of cells of the *Dinophysis acuminata*-complex.

Cells of *Dinophysis caudata* and *D. acuminata*-complex (two different morphotypes *D*. cf. *acuminata* and *D*. cf. *ovum*) (Figure 3) were isolated one by one from the plankton net-haul concentrates with a microcapillary pipette under an inverted microscope Olympus IX71, at 200× magnification. Each picked cell was transferred three times through drops of sterile seawater and finally placed (with as little seawater as possible) in a 1.5 mL Eppendorf tube with 500 µL methanol, and kept at −20 °C until analysis.

### 5.3. Standards and Reagents

LC-MS grade methanol (MeOH) and acetonitrile (CH_3_CN) were used for the extraction and analyses of toxins by liquid chromatography coupled to mass spectrometry (LC-MS). Analytical-grade ammonium hydroxide, sodium hydroxide (NaOH), and hydrochloric acid (HCl) were used for the mobile phase and hydrolysis. Ultrapure water was obtained with a Sartorius (Arium Pro) purification system. Certified reference solutions for okadaic acid CRM-OA-d (batch #20141119), dinophysistoxin-2 CRM-DTX2-b (batch #20150819), dinophysistoxin-1 CRM-DTX1-b (batch #20151209), and pectenotoxin-2 CRM-PTX2-b (batch #20120516) were obtained from NRC-CNRC.

### 5.4. Toxins Extraction

#### 5.4.1. From Isolated Cells of *Dinophysis*

To prepare for LC/MS analysis, samples kept frozen in Eppendorf tubes were transferred to a 2-mL microtube, the remains in the Eppendorf tube were washed twice with 200 µL of methanol, incorporated to the microtube, and mixed in a vortex prior to being dried at 40 °C under a flow of nitrogen gas. The dried toxin extract was re-suspended in 500 µL of methanol, mixed in a vortex, and filtered through 0.2 µm pore size nylon filters (Sterlitech, 13 mm) as described in [23].

#### 5.4.2. From Shellfish Meat

Whole flesh samples of 12–15 scallops, *Argopecten purpuratus,* were homogenized and a 2 ± 0.05 g subsample, weighed on an analytical scale (Precisa, LX 220A), was placed in a 50 mL centrifuge tube and extracted twice with 9 mL methanol, stirred with a vortex (Thermo, maxi mix II) for 3 min, and centrifuged at 2000× *g* (Thermo, Sorvall ST 16R) for 10 min at a temperature of 20 °C. The supernatants were transferred and mixed in a volumetric flask and made up to 20 mL with methanol. To explore the presence of esterified derivatives of OA and DTXs, an aliquot of the methanolic extract was taken for alkaline hydrolysis. 

The alkaline hydrolysis was carried out by adding 125 µL of 2.5 N NaOH to 1 mL of the methanolic extract, vortexing the mixture for 0.5 min, heating it for 40 min at 76 °C, and finally neutralizing the added NaOH with an equivalent amount of 2.5 N HCl. 

Finally, all extracts (raw and hydrolyzed) were filtered through a 0.2 µm pore size nylon filter (Chromafil^®^Xtra, 25 mm) following the recommended protocols from the European Union Reference Laboratory for Marine Biotoxins [24].

### 5.5. LC-MS/MS Analyses

For LC-MS/MS analysis of the lipophilic toxins, a Waters Acquity I Class chromatograph coupled to a triple quadrupole mass spectrometer Waters XEVO-TQS by means of an electrospray interface (ESI) was used. Analytical separation was performed following a modification of the method developed by Gerssen et al. [25], with an Acquity UPLC^®^ BEH C18 (1.7 µm, 2.1 × 100 mm) column kept at 40 °C. A binary gradient elution was used, with phase A consisting of H_2_O and phase B of 90% CH_3_CN, both containing ammonium hydroxide 6.7 mM (approximate pH of 11). The gradient started with 30% B, that proportion was kept for 1 min and then linearly increased to 90% B in 4 min. It was maintained at that proportion for 1 min, returned to the initial proportion in 0.1 min, and maintained for equilibration during 1.5 min before the next injection. The flow rate was 0.4 mL min^−1^ and the injection volume was 2 µL. The mass spectrometer was operated in both ESI positive and negative modes, the cone voltage was 3.0 kV, desolvation gas temperature was 500 °C with an N_2_ flow of 1000 L h^−1^ and a source temperature of 150 °C. Voltage parameters of the cone and collision energy were optimized during the tuning phase by direct infusion in alkaline medium. Product ions used for the quantification of each toxin in microalgae and shellfish and the MS/MS conditions for the multiple reaction monitoring (MRM) for each molecule are shown in Table 3.

## Figures and Tables

**Table 1:** Toxin content in isolated cells of *Dinophysis*.

Date (d/m/y)	Location	Sampling Depth (m)	Species	Picked Cells (No.)	OA (pg Cell^−1^)	PTX2 (pg Cell^−1^)
27/01/2017	Independencia Bay	0–8	*D. acuminata*-complex	150	<LOD *	9.6
09/02/2017	Samanco Bay	0–15	*D. caudata*	92	<LOD *	9.2
10/04/2017	Salinas	0–15	*D. acuminata*-complex	204	<LOD *	9.3
24/03/2018	Sechura Bay (Vichayo)	0–15	*D. acuminata*-complex	440	0.8	11.1
24/03/2018	Sechura Bay (Puerto Rico)	0–15	*D. acuminata*-complex	400	0.3	1.5
14/04/2018	Sechura Bay (Parachique)	0–15	*D. caudata*	400	<LOD *	5.8

* LOD: 0.16 ng mL^−1^. OA: okadaic acid; PTX: pectenotoxin.

**Table 2:** Toxin content in *Argopecten purpuratus* (whole flesh).

Date d/m/y	Place	OA µg kg^−1^ Post-Hydrolysis	PTX2 µg kg^−1^
27/01/2017	Independencia Bay-El Queso	<LOD *	22.2
09/02/2017	Samanco Bay	<LOD *	20.3
10/04/2017	Salinas	<LOD *	10.3
14/04/2018	Sechura Bay-Puerto Rico	10.4	34.8
14/04/2018	Sechura Bay-Barrancos	8.6	20.8
14/04/2018	Sechura Bay-San Pedro	15.2	27.6
05/05/2018	Sechura Bay-Las Delicias	7.7	10.7

* LOD: 1.6 µg kg^−1^.

**Table 3:** Multiple reaction monitoring (MRM) and MS/MS of each toxin from the shellfish analysis.

Toxins	ESI Mode	Ion		Cone Voltage (V)	Collision Energy (CE) (eV)	Dwell (s)
		Precursor (*m*/*z*)	Product (*m*/*z*)			
OA	ESI^−^	803.5	255.1 *	30	50	0.05
			113.0		60	
DTX1	ESI^−^	817.5	255.1 *	30	50	0.05
			113.0		60	
DTX2	ESI^−^	803.5	255.1 *	30	50	0.05
			113.0		60	
PTX1	ESI^+^	892.5	821.5 *	30	30	0.02
			213.3		40	
PTX2	ESI^+^	876.6	823.5 *	30	20	0.20
			213.1		40	

* Transitions used for the phytoplankton analyses. ESI: electrospray ionization.
